# The F-box protein MAX2 contributes to resistance to bacterial phytopathogens in *Arabidopsis thaliana*

**DOI:** 10.1186/s12870-015-0434-4

**Published:** 2015-02-13

**Authors:** Maria Piisilä, Mehmet A Keceli, Günter Brader, Liina Jakobson, Indrek Jõesaar, Nina Sipari, Hannes Kollist, E Tapio Palva, Tarja Kariola

**Affiliations:** Division of Genetics, Department of Biosciences, Faculty of Biological & Environmental Sciences, University of Helsinki, Helsinki, FIN-00014 Finland; Austrian Institute of Technology GmbH, Bioresources, Health and Environment Department, Tulln an der Donau, 3430 Austria; Viikki Metabolomics Unit, Department of Biosciences, Faculty of Biological and Environmental Sciences, University of Helsinki, Helsinki, FIN-00014 Finland; Institute of Technology, University of Tartu, Nooruse 1, Tartu, 50411 Estonia

**Keywords:** *Arabidopsis thaliana*, F-box proteins, ROS, Ozone, Phytopathogen, *P. syringae*, *P. carotovorum*, Stomata, Plant defense, ABA, SA

## Abstract

**Background:**

The *Arabidopsis thaliana* F-box protein MORE AXILLARY GROWTH2 (MAX2) has previously been characterized for its role in plant development. MAX2 appears essential for the perception of the newly characterized phytohormone strigolactone, a negative regulator of polar auxin transport in Arabidopsis.

**Results:**

A reverse genetic screen for F-box protein mutants altered in their stress responses identified MAX2 as a component of plant defense. Here we show that MAX2 contributes to plant resistance against pathogenic bacteria. Interestingly, *max2* mutant plants showed increased susceptibility to the bacterial necrotroph *Pectobacterium carotovorum* as well as to the hemi-biotroph *Pseudomonas syringae* but not to the fungal necrotroph *Botrytis cinerea. max2* mutant phenotype was associated with constitutively increased stomatal conductance and decreased tolerance to apoplastic ROS but also with alterations in hormonal balance.

**Conclusions:**

Our results suggest that MAX2 previously characterized for its role in regulation of polar auxin transport in Arabidopsis, and thus plant development also significantly influences plant disease resistance. We conclude that the increased susceptibility to *P. syringae* and *P. carotovorum* is due to increased stomatal conductance in *max2* mutants promoting pathogen entry into the plant apoplast. Additional factors contributing to pathogen susceptibility in *max2* plants include decreased tolerance to pathogen-triggered apoplastic ROS and alterations in hormonal signaling.

**Electronic supplementary material:**

The online version of this article (doi:10.1186/s12870-015-0434-4) contains supplementary material, which is available to authorized users.

## Background

Phytohormones are central regulators of all aspects of plant life. They modulate plant development and reproduction as well as regulate responses to both biotic and abiotic environmental stresses, which are a constant challenge to plant growth and survival. Different stresses trigger distinct signaling pathways: abscisic acid (ABA) is a central mediator of responses to abiotic stresses whereas salicylic acid (SA), jasmonates (JA) and ethylene (ET) signaling mediate responses to invading pathogens [1-3]. A central component in phytohormone-mediated stress and defense signaling is the modulation of stomatal function. Stomata regulate the gas exchange of plants by rapidly responding to environmental signals such as light, CO_2_ level and changing concentrations of phytohormones [4-6]. While in response to various abiotic stresses such as drought the role of ABA is central in promoting stomatal closure [7] in pathogen-triggered innate immunity responses this process also requires SA [6,8,9]. Importantly, several studies have shown that many foliar phytopathogens take advantage of stomata as natural openings when entering the plant and consequently plant mutants with more open stomata often show enhanced susceptibility to pathogens [6]. The recognition of PAMPs (pathogen associated molecular patterns), such as bacterial flagellin, triggers stomatal closure, which is a central part of the innate immune response in Arabidopsis [6].

Different hormonal pathways share both synergistic and antagonistic crosstalk. This communication is not only essential in order to reach the most efficient signaling and signal fine-tuning but also in defining the response priorities to avoid the wasting of limited resources of the plant [2,10]. For example, SA-, JA- or ABA-mediated signaling pathways triggered by stress can be further modulated by other phytohormones. The role of auxin is well-characterized in plant growth and development but yet, it is also long known to antagonize the ABA-induced stomatal closure [11]. Furthermore, auxin has been shown to influence stomatal function by promoting stomatal opening and thus, enhancing the progression of pathogen infection [12-14]. Additionally, auxin signaling was shown to increase disease symptoms by pathogens such as *Botrytis cinerea* and *Pseudomonas syringae* [13,15] and several auxin signaling mutants demonstrate increased tolerance to different pathogens [16-20]. The antagonistic impact auxin has on SA is well characterized [3,13]. At the same time, SA-mediated defenses often repress auxin signaling, demonstrated as down-regulation of small auxin-up RNA (SAUR) genes, Aux/IAA genes, and auxin receptor genes as well as genes related to polar auxin transport [14]. Thus, modulation of endogenous hormone levels can considerably influence the stomatal movement and hormone signaling balance and hence, the outcome of pathogen infection.

While having different roles in plant defense, signaling pathways also share similar elements. Activation of defense signaling in response to both abiotic and biotic stress involves production of reactive oxygen species (ROS). For example, both pathogen infection and ozone cause ROS production in the apoplastic space of the plant cell which further induces stress tolerance and acclimation via a wide range of signaling events [21-23].

F-box proteins are central regulatory components in many of the hormonal pathways [24]. In Arabidopsis, there are 700 F-box proteins but many still remain without an assigned function [25]. Among the well-characterized examples are the auxin receptor TIR1 (TRANSPORT INHIBITOR REPONSE1) [26] and the jasmonate receptor COI1 (CORONATINE INSENSITIVE1) [27]. One of the proteins characterized for its influence on endogenous auxin balance in Arabidopsis is MAX2 (MORE AXILLARY GROWTH2) [28,29]. MAX2 is a member of the F-box leucin-rich repeat family of proteins, a component of the SCF complex acting in the ubiquitin proteasome pathway that via ubiquitination marks proteins for destruction by the 26S proteasome [24,30]. Intriguingly, the impact of MAX2 on plant auxin status is mediated via the proposed perception of a newly discovered phytohormone, strigolactone [31-34], first identified as a germination stimulant for parasitic plants of the genera *Orobanche* and *Striga* (hence the name strigolactone) [35,36]. The plant-produced strigolactones secreted from roots can stimulate plant interactions with arbuscular mycorrhizal fungi [35,37]. The impact of strigolactones on plant auxin status is negative i.e. they influence polar auxin transport to control branching. MAX2, proposed to act in strigolactone perception, participates in an SCF complex that locally suppresses shoot branching and accordingly, the shoot branching phenotype of *max* mutants is caused by increased auxin transport capacity in the main stem [29,38,39]. Other *MAX*-genes of the pathway, *MAX1, MAX3* and *MAX4*, are associated with the biosynthesis of strigolactones, terpenoid lactones derived from the carotenoid pathway [31-33]. Interestingly, the role of MAX2 expands further than just the involvement in strigolactone signaling and the regulation of auxin transport: recently it was shown to be essential in karrikin signaling [40]. Karrikins are allelochemicals found in smoke that act by promoting seed germination and hence, they influence the early development of many plants by an unknown mechanism [40,41].

Our interest lies in the characterization of plant response to pathogens and thus, we established a reverse genetics screen of a number of yet uncharacterized F-box T-DNA mutant lines in order to find new, stress-related phenetypes. To accomplish this, we screened the mutants for their ozone sensitivity. Ozone exposure provides a convenient and robust tool to screen for mutants with altered stress tolerance, and plant responses to ozone and pathogens share common elements such as ROS burst via the activation of apoplastic NADPH oxidase [21,42-44]. One of the F-box protein mutants with clearly increased ozone susceptibility harbored a T-DNA insertion in the *MAX2* gene previously characterized for its role in strigolactone perception and thus, negative regulation of polar auxin transport [29]. Interestingly, further characterization revealed that MAX2 is required for proper stomatal function in response to ozone, CO_2_ and ABA, and that the corresponding gene is also required for full resistance to pathogens in Arabidopsis. Furthermore, MAX2 appeared to contribute to defense against two bacterial phytopathogens with different lifestyles: *max2* mutant lines demonstrated increased susceptibility to the hemibiotroph *Pseudomonas syringae* and to the necrotroph *Pectobacterium carotovorum*. This phenotype was suggested to result from more open stomatal aperture accentuated by increased sensitivity to apoplastic ROS and alterations in endogenous phytohormone signaling.

## Results

### F-box protein MAX2 is required for ozone tolerance in Arabidopsis

To identify F-box genes involved in plant responses to environmental stresses we screened a collection of 60 T-DNA insertion lines from the F-box protein families C1, C2, C3 and C4 (according to classification by Gagne et al. 2002 [25]) for altered stress responses. This particular group was chosen since it contains, apart from many proteins with unknown function, also the proteins TIR1, COI1 and EBF1 and EBF2 (EIN3 BINDING F-BOX1 and 2) already characterized to be centrally involved in plant hormone signaling [45-47]. As a positive control we used *rcd1-4* (*radical-induced cell death1*) mutant plants which have a well-characterized ozone sensitive phenotype [42].

**Figure 1:**
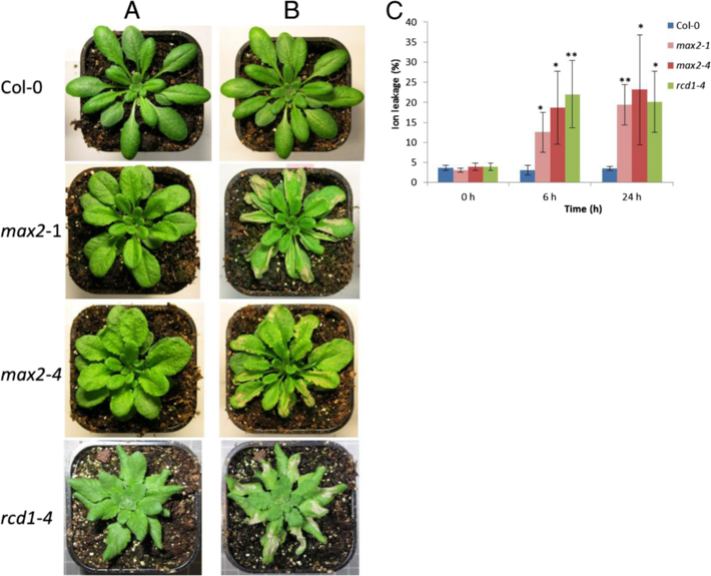
***max2*** **plants are highly susceptible to ozone.** Soil grown four weeks old wild-type Col-0, *max2* point mutation (*max2-1*), Salk (*max2-4*) and *rcd1-4* (as an ozone sensitive control) lines were exposed to 350 ppb ozone (O_3_) for 6 h in a controlled O_3_ chamber. The plants were photographed before and 1 day after O_3_ exposure in order to show cell death on the leaves. **A)** Non-treated Col-0, *max2-1*, *max2-4* and *rcd1-4* lines grown in clean air. **B)** O_3_ phenotype of Col-0, *max2-1*, *max2-4* and *rcd1-4* lines 1 day after 6 h O_3_ exposure. **C)** Ion leakage in Col-0, *max2-1*, *max2-4* and *rcd1-4* lines measured at different time points after O_3_ exposure indicating the amount of cell death. The result is presented as ratio of ion leakage of total ion concentration. Data represent the means ± SE of 3 independent experiments with 5 plants/line in every time point in each experiment. **P < 0.01; two-tailed t test.

After 6 h exposure to 300 ppb of ozone *rcd1-4* plants had developed distinct lesions while wild-type plants did not show any signs of damage (Figure 1). Of the tested F-box T-DNA lines, approximately 10 displayed varying degree of lesion formation. The line with the most distinct increase in ozone sensitivity*, max2-4* (SALK_028336)*,* harbored a T-DNA insertion in a gene encoding the F-box protein MAX2 (MORE AXILLARY GROWTH2), previously well characterized as a negative regulator of polar auxin transport [29]. Interestingly, in response to ozone, the *max2-4* plants developed clearly visible and spreading lesions (Figure 1A and B). Increased ozone sensitivity was observed also for *max2-1* point mutation line [28] confirming that the phenotype was indeed a result from mutation in the *MAX2* gene (Figure 1A and B). The observed ozone sensitivity was further confirmed by measuring the ion leakage from the *max2* mutants and wild-type plants at time points 0, 8 and 24 h after beginning of ozone exposure (Figure 1C). In *max2* mutants the ion leakage was clearly higher compared to wild-type plants. These results strongly indicate that MAX2 contributes to ozone tolerance in Arabidopsis.

### MAX2 provides tolerance to apoplastic O_2_•^−^

Production of reactive oxygen species (ROS) is a common response to environmental stresses in plants and accordingly, also ozone is known to trigger the production of superoxide (O_2_•^−^ ) in the apoplastic space of plant cells leading to formation of visible lesions in sensitive plants [42]. Since the impaired function of MAX2 had led to increased ozone sensitivity, we wanted to further investigate the contribution of ROS in the observed lesion formation in *max2* plants. To address this we employed the extracellular O_2_•^−^ generating system, xanthine (X)/xanthine oxidase (XO) [42,48]. We infiltrated the leaves of wild-type and *max2* plants with X/XO and the resulting cell death was measured as relative ion leakage and monitored for 24 h. Again, *rcd1-4* plants that are known to be sensitive to extracellular ROS [42] were included as positive controls.

**Figure 2:**
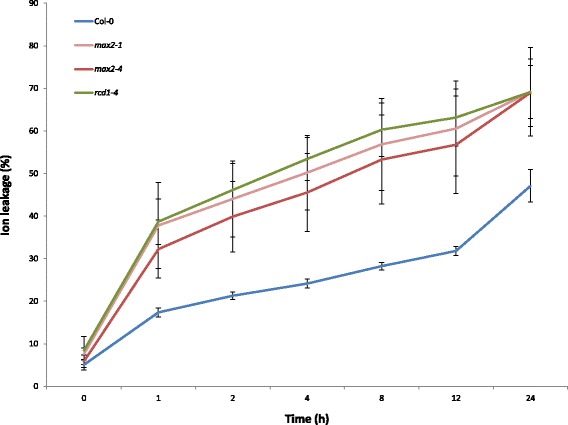
**Superoxide (O**_**2**_ **•** ^**−**^ **) induced cell death in** ***max2*** **mutants.** Detached leaves from four week old soil grown wild-type Col-0, *max2-1*, *max2-4* and *rcd1-4* mutant plants were infiltrated with the O_2_•^−^ generating system xanthine and xanthine oxidase (X/XO). Cell death was measured as relative ion leakage for 24 h. Data are means ± SE from 3 independent experiments with >20 leaves/line in each experiment. The result is presented as ratio of ion leakage of total ion concentration.

Interestingly, in accordance with the observed sensitivity to ozone (Figure 1), the accumulation of O_2_•^−^ led to increased ion leakage in both *max2* mutant lines in comparison to wild-type (Figure 2). In X/XO-infiltrated *max2* mutant lines the ion leakage increased 25% during the first hour while in wild type the corresponding increase was 15% (Figure 2). Increase in ion leakage was even more distinct during the next 12 h. Since X/XO –experiment is done by infiltrating and thus, is independent of stomatal opening, it seems that MAX2 influences plant sensitivity to ROS in the level of mesophyll.

To further characterize the nature of the decreased ROS tolerance observed in *max2* mutant lines, we tested if these lines were also more sensitive to methyl viologen that generates ROS inside the chloroplasts. Also here, *rcd1-4* plants tolerant to methyl viologen were included as controls [49]. However, no difference was observed in the methyl viologen tolerance of *max2* mutant lines in comparison to wild-type plants (Additional file 1: Figure S1). Thus, these results indicate that MAX2 specifically contributes to apoplastic O_2_•^−^ tolerance in Arabidopsis.

### MAX2 influences stomatal conductance in Arabidopsis

**Figure 3:**
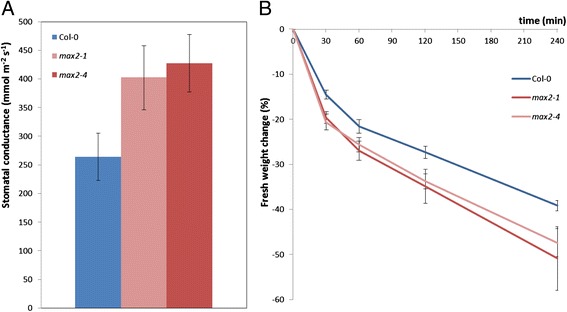
**Impaired stomatal function in** ***max2*** **mutants.** Four-week old wild-type Col-0 and *max2* lines were assessed for their stomatal function. **A)** Stomatal conductance of four-week old non-treated Col-0, *max2-1* and *max2-4* plants were measured with a porometer. For each line 5 plants were used in each experiment and the results are shown as means ± SE. Experiments were repeated 5 times with similar results. **P < 0.01; two-tailed t test. **B)** Four-week old soil-grown Col-0 and *max2* plants’ fresh weight change was measured by cutting the leaves and leaving them to dry for 4 h. For each line 5 plants were used in each experiment and the results are shown as means ± SE. Experiments were repeated 5 times with similar results.

Both ozone as well as pathogens can enter the plant apoplast via natural openings such as stomata [6,42]. We hypothesized that besides increased sensitivity to apoplastic ROS, the sensitivity of *max2* plants to ozone could be partly due to altered stomatal function. To validate this hypothesis we first measured stomatal conductance of *max2* and wild-type plants with a porometer. Indeed, under normal growth conditions the stomata of *max2* mutant plants were significantly more open in comparison to those of wild-type plants (Figure 3A).

Additionally, we monitored the fresh weight change of excised leaves, which also reflects the amount of gas exchange and water loss from the plant to the atmosphere. This was done by comparing the weight change in wild-type and *max2* mutant plants for 4 h. In concert with the results from the porometer measurement, the percentual fresh weight loss of *max2* plants was significantly larger than that of wild-type plants (Figure 3B), which further indicates a role for MAX2 in stomatal regulation.

**Figure 4:**
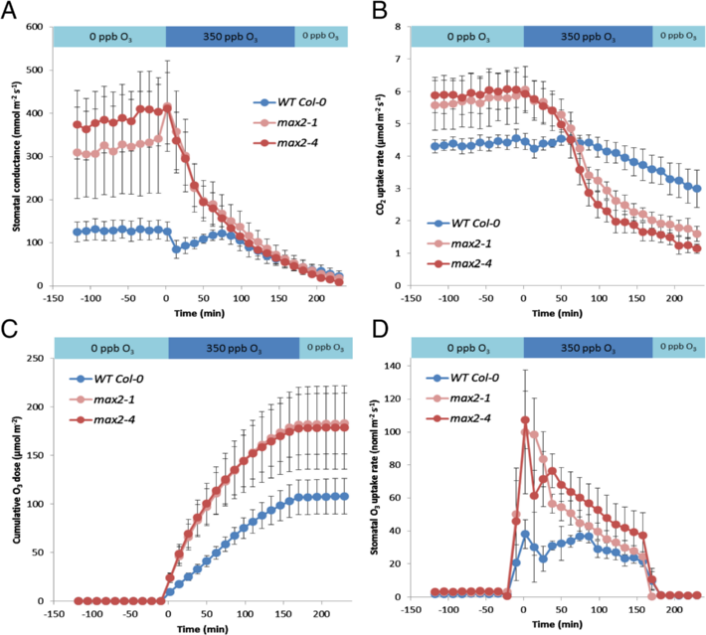
**MAX2 controls the basal level of stomatal conductance.** Effects of 3 h 350 nmol/mol O_3_ exposure on stomatal conductance were measured on wild-type Col-0 and *max2* mutants with a custom made whole-rosette gas exchange measurement device. **A)** Stomatal conductance before, during and after 3 h O_3_ exposure of Col-0 and *max2* plants. **B)** CO_2_ uptake rate of *max2* mutants and Col-0 before, during and after 3 h O_3_ exposure. **C)** Cumulative dose of O_3_ absorbed by *max2* and Col-0 plants before, during and after 3 h O_3_ exposure. **D)** Stomatal O_3_ uptake rate of *max2* mutants and Col-0. For each line 4 plants were used in the experiment and the results are shown as means ± SE. Experiments were repeated twice with similar results.

The enhanced stomatal conductance of *max2* mutants was verified by measuring stomatal conductance of non-treated and ozone exposed *max2* plants with a custom made gas-exchange device [50]. In agreement with the porometer measurement, the basal level of stomatal conductance before the ozone exposure was two times higher in the *max2* mutant lines than that observed in wild-type plants (Figure 4A). However, the application of O_3_ (in time point 0 min) induced rapid stomatal closure in both *max2* mutant and wild-type plants. Interestingly, a slight recovery of stomatal conductance was observed after the closure in wild-type plants, but not in *max2* plants (Figure 4A). This could be explained by the rapid, O_3_-triggered induction of cell death in *max2* mutants, further supported by the quick decrease of general photosynthesis (CO_2_ uptake, μmol/m^2^s) in these plants (Figure 4B). While the ozone-induced stomatal closure of *max2* plants was as rapid as that detected in wild-type plants (Figure 4A), the intake of ozone still remained higher (Figure 4C) due to the higher stomatal conductance at the beginning of the ozone exposure. Stomatal O_3_ uptake rate of *max2* mutants was higher compared to Col-0 (Figure 4D) probably due to more open stomata.

ABA is a well-known regulator of stomatal closure and plant drought responses [51]. The more open stomata as well as increased water-loss of the *max2* plants (Figure 3A and B) suggested alterations in ABA-reponses and thus, it was of interest to elucidate if the stomatal response to this phytohormone was altered in these plants. Interestingly, this was not the case since *max2* plants displayed wild-type stomatal closure in response to 5 μM ABA sprayed onto intact plants (Additional file 1: Figure S2) indicating that at whole plant level MAX2 contributes to the basal level of stomatal conductance rather than to stomatal closure induced by ABA and ozone.

### MAX2 contributes to resistance to bacterial, but not fungal pathogens

**Figure 5:**
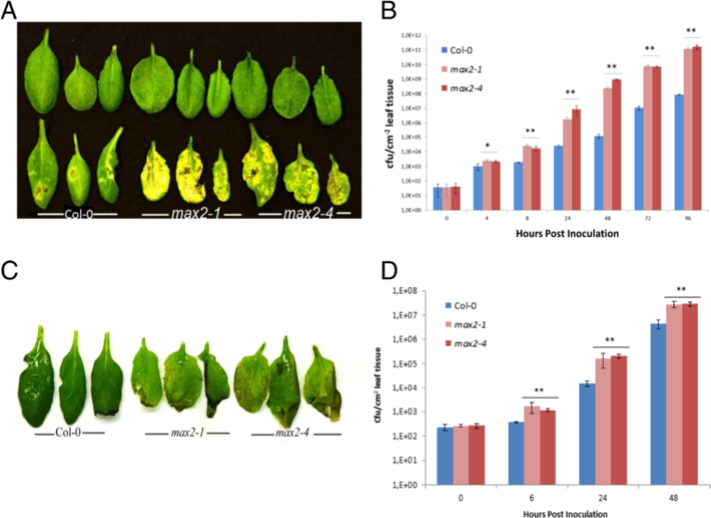
***max2*** **mutant lines have decreased resistance to spray inoculated** ***P. syringae*** **and** ***P. carotovorum*** **.** Soil-grown four-week old plants were used to evaluate pathogen tolerance. In each experiment, three plants/line and three leaves/plant were used to check phenotype and to measure the bacterial concentration. All the experiments were repeated at least 4 times with similar results. The results are shown as means ± SE. (*P < 0.05; **P < 0.01; two-tailed t test). **A)** Phenotype of four week old wild-type Col-0 and *max2* mutants after *P.syringae* infection with the concentration of 1x10^7^ cfu/ml. Picture was taken 5 days post inoculation. Upper row shows non-treated plants and lower row *P. syringae* infected plants. **B)** Growth of *P. syringae* in planta was calculated at 0, 4, 8, 24, 48, 72 and 96 h after inoculation. **C)** Phenotype of *max2* mutant lines after infection with *P.carotovorum*. Picture was taken 2 days post inoculation. **D)** Growth of *P. carotovorum* in planta 0, 6, 24 and 48 h after infection.

The clearly altered stomatal phenotype implied that impaired expression of *MAX2* gene could have an impact on pathogen tolerance in Arabidopsis. To elucidate this, we first investigated the susceptibility of *max2* mutant lines to the virulent bacterial hemibiotroph *P. syringae* DC3000. To this aim we spray-inoculated *max2* mutant lines and wild-type plants with the pathogen and followed the symptom development and bacterial growth *in planta* for five days. Interestingly, *max2* mutant plants displayed clearly enhanced susceptibility to *P. syringae* observed both as heavy yellowing of the infected leaves as well as increased growth of the bacteria in the apoplast (Figure 5A and B). To further define the role of MAX2 in pathogen responses, we employed another type of pathogen, a bacterial necrotroph *P. carotovorum*, the causal agent of bacterial soft rot [52,53]. Interestingly, spray inoculation of the plants with *P. carotovorum* also resulted in enhanced disease development in the *max2* mutant lines seen as more extensive tissue maceration when compared to wild-type plants (Figure 5C and D) indicating that the defense-associated role of MAX2 is not dependent on the pathogen lifestyle.

To assess if the infection method had any impact on the observed disease phenotype, we did local inoculations with *P. syringae* (infiltration) and *P. carotovorum* (pipetting the bacterial solution to wounded leaf), thereby providing the bacteria a direct route to plant apoplast. Intriguingly, when *P. syringae* was applied by infiltration method, slightly enhanced susceptibility was still observed in *max2* mutant lines. The same was observed after infection with *P. carotovorum, max2* plants demonstrated slight increase in the susceptibility in comparison to wild-type (Additional file 1: Figure S3). The distinct difference observed in pathogen susceptibility resulting from the different inoculation methods indicated that the stomatal phenotype of *max2* plants (Figure 3A) has a central impact on the outcome of the infection i.e. more open stomata of *max2* mutant plants increase bacterial entry to the apoplast of these plants.

The evident contribution of MAX2 in resistance to bacterial pathogens prompted us to elucidate whether this was also the case in plant defense to fungal pathogens. To test this, we infected *max2* and wild-type plants with *Botrytis cinerea*, a fungal necrotroph and followed the symptom development for three days. Interestingly, opposite to observations with *P. carotovorum* and *P. syringae*, no difference could be observed in susceptibility between the *max2* lines and wild-type plants for *B. cinerea* (Additional file 1: Figure S4). This indicates that the difference observed in the susceptibility of *max2* lines to different pathogens results from the enhanced capability of the bacterial pathogens to take advantage of the impaired stomatal function of *max2* lines (Figures 3A and 4) when entering the plant apoplast.

### MAX2 is required for pathogen-triggered stomatal closure

**Figure 6:**
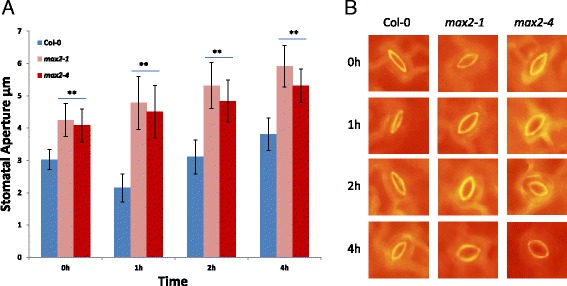
**Pathogen-triggered stomatal closure is impaired in max2 mutant lines.** Four-week old wild-type Col-0 and *max2* lines were inoculated with *Pseudomonas syringae* pv. *tomato* DC3000. **A)** Measurement of stomatal aperture of wild-type Col-0 and *max2* lines in response to *P. syringae*. Leaves were first stained with 20 μM propidium iodide (PI) solution and then inoculated with 300 μl of bacterial solution (10^8^ cfu/ml). Stomatal aperture width was measured after indicated time points using ImageJ image processing program. **B)** Representative pictures of stomatal response of Col-0 and *max2* lines under florescent microscope using 20x objective 0, 1, 2 and 4 h after inoculation with the bacteria. Results are shown as the mean (n = 80-100) ± SE. **P < 0.01; two-tailed t test. The experiments were repeated three times with similar results.

Stomatal closure in response to invading bacteria such as *P. syringae* is a well-described component of the innate immunity response in Arabidopsis [6]. The more open stomatal aperture in the absence of stress (Figure 3A) and the enhanced susceptibility of *max2* plants to spray-inoculated *P. syringae* (Figure 5A and B) indicated that the pathogen-triggered stomatal closure could be impaired in these plants. To elucidate this, we infected *max2* and wild-type plants with *P. syringae* bacterial suspension and checked stomatal response to living bacterial cells 0, 1, 2 and 4 h after inoculation using fluorescence microscopy using the method introduced by Chitrakar and Melotto 2010 [54]. When *max2* and wild-type leaves were incubated with *P. syringae* stomatal closure was triggered in wild-type plants1h after infection but this was not observed in *max2* lines where the stomatal opening was rather getting higher during measured time points (Figure 6). *P. syringae* DC3000 has been shown to induce re-opening of the stomata from 3 to 4 h after the initial closure by secreting the phytotoxin coronatine [6]. While this was observed for the wild-type at 4 h time point, in *max2* plants the stomatal aperture was even larger 2 and 4 h after the infection (Figure 6). Treatment of *max2* and wild-type leaves with MgCl_2_ buffer solution did not alter the stomatal aperture, but yet, the stomata of *max2* plants were clearly more open compared to wild-type (Additional file 1: Figure S5). These results clearly indicate that *max2* plants have impaired stomatal closure in response to *P. syringae* allowing increased numbers of bacteria to enter plant apoplast (Figure 5B) leading to more severe susceptibility.

### *max2* plants exhibit increased expression of genes triggered by oxidative stress in response to ozone and *P. syringae*

**Figure 7:**
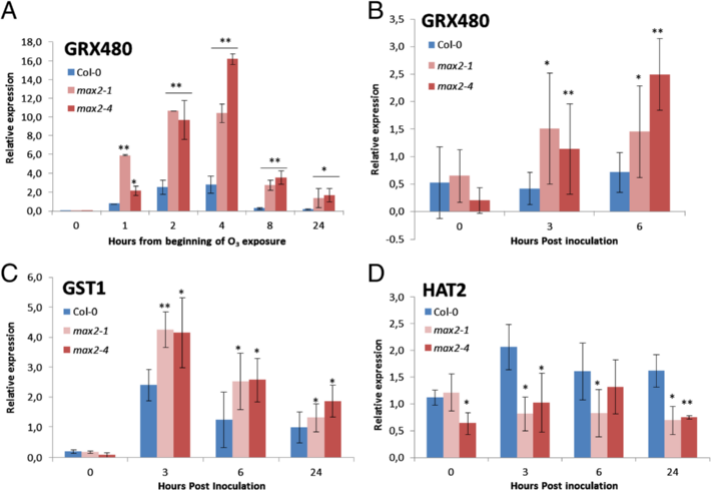
**The expression of oxidative stress marker genes in** ***max2*** **lines is upregulated.** Mature leaves of 4-week old soil grown wild-type Col-0 and *max2* plants were collected at indicated time points after *P. syringae* DC3000 infection and RNA was extracted to check the relative expression of oxidative stress marker gene *GRX480* after 350 ppb for 6 h ozone exposure **(A)** and after pathogen infection **(B)**. Another oxidative stress marker gene *GST1* **(C)** and auxin-responsive gene *HAT2* **(D)** also checked after pathogen infection. For this analysis, 3 plants/line and 3 leaves/plant were used in each time point of infection and ozonation. Each expression analysis is based on a minimum of 3 independent experiments. Asterisks indicate significant differences, as determined by Student’s t-test (*P < 0.05; **P < 0.01; two-tailed t test).

The enhanced sensitivity of *max2* mutants to apoplastic ROS (Figure 2) suggested that MAX2 could be involved in responses to oxidative stress. Since both ozone and pathogen infection trigger apoplastic ROS formation, we wanted to study the induction of ROS-responsive genes in *max2* and wild-type plants in response to these stresses. For this, we first characterized the expression of *GRX480* encoding a glutaredoxin family protein, that is an early ROS responsive gene and is also triggered by ozone [55,56]. Ozone triggered overall higher expression of *GRX480* than *P. syringae* but in both cases the induction of this gene was clearly higher in *max2* than in wild-type plants (Figure 7A and B). We also characterized the expression of oxidative stress marker gene *GST1* (*ARABIDOPSIS GLUTATHIONE S TRANSFERASE1*) [57] in response to *P. syringae*. Similarly to *GRX480* the accumulation of *GST1* transcripts was also enhanced in *max2* plants when compared to wild-type plants but to higher level (Figure 7C). These observations suggest that *max2* plants might be more sensitive to ROS and that MAX2 is involved in oxidative stress responses.

Moreover, the expression of *HAT2*, an auxin-responsive homeobox-leucine zipper gene has been shown to decrease in response to ozone-triggered ROS [55]. Therefore, it was of interest to check if the expression of this gene was altered in *max2* plants where auxin homeostasis was modulated and same was indicated for ROS responses (Figure 7D). Similarly to Blomster et al. 2011 [55] the levels of *HAT2* were decreased in wild-type plants in response to ozone and it was even slightly lower in *max2* in the early timepoints (Additional file 1: Figure S6). However, *P. syringae* triggered expression of *HAT2* was clearly lower in *max2* when compared to wild-type plants (Figure 7D). The decreased induction of this gene in *max2* plants might be an indication of altered responsiveness to apoplastic ROS in these plants.

The expression of the ROS responsive genes *GRX480*, *GST1* and *HAT2* suggested that the sensitivity to apoplastic ROS might have altered in *max2* plants. Therefore, we wanted to further clarify whether MAX2 indeed influences the the sensitivity to or rather the cellular level of ROS we performed O_2_•^−^ and H_2_O_2_ stainings after ozone exposure and *P. syringae* infection. These semiquantitative stainings did not reveal visible differences between wild-type and *max2* mutant lines (data not shown). The lack of enhanced ROS production further underlines that the enhanced gene expression triggered by oxidative stress is likely to be due to altered ROS-sensitivity in *max2* plants.

### Expression of auxin receptor genes is downregulated in *max2* plants

**Figure 8:**
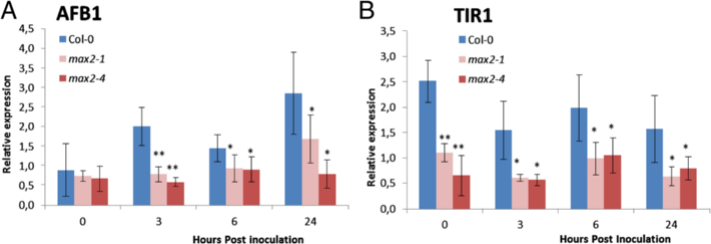
**Expression of auxin marker genes in** ***max2*** **lines are downregulated.** Leaves from 4-week old soil grown wild-type Col-0 and *max2* line plants were collected at indicated time points after *P. syringae* DC3000 infection and used to extract RNA to check the relative expression of auxin marker genes, *AFB1* **(A)** and *TIR1* **(B)**. For this analysis, 3 plants/line and 3 leaves/plant were used. Results are based on a minimum of 3 independent experiments. Asterisks indicate significant differences, as determined by Student’s t-test (*P < 0.05; **P < 0.01; two-tailed t test).

MAX2 has been shown to negatively regulate polar auxin transport in Arabidopsis i.e. auxin transport is increased in *max2* mutants [29]. Furthermore, the expression of *SAUR*-genes is enhanced in *max2* plants indicating increased auxin response [58]. Auxin homeostasis has been shown to influence some plant-pathogen interactions [59] and interestingly, also *max2* mutants were more sensitive to phytopathogens than wild-type plants. Thus, we wanted to explore whether auxin-related gene expression was also altered in *max2* plants in response to *P. syringae* DC3000. Suprisingly, we noticed that the expression of the auxin receptor genes *AUXIN SIGNALING F-BOX PROTEIN1 (AFB1)* and *TRANSPORT INHIBITOR RESPONSE1 (TIR1)* was altered in *max2* lines in comparison to wild-type plants. While *P. syringae* triggered *AFB1* induction in wild-type plants, this was not observed in *max2* plants (Figure 8A). Furthermore, *TIR1* expression was decreased in *max2* plants already before pathogen inoculation and remained in significantly lower level than in wild-type during the course of infection (Figure 8B). This could reflect the attempt of the plant to reduce the increased auxin response by downregulating the expression of the corresponding receptors.

### Phytohormone levels are altered in *max2* mutant plants

**Figure 9:**
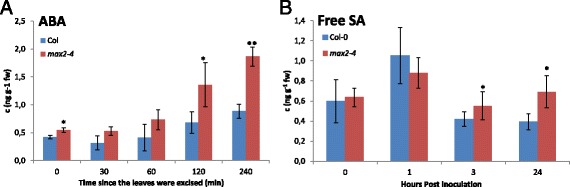
**Altered phytohormone levels in Col-0 and** ***max2*** **mutant lines.** Hormone levels in *max2* mutant plants were measured in response to both drought (excised leaves) for ABA and pathogen infection (*P. syringae* DC3000) for SA. The results shown are representative of both *max2* mutant lines. **A)** ABA levels of *max2* mutant plants in response to drought. The values are means ± SE of 2 independent experiments with 3 biological repeats in each experiment. Asterisks indicate significant differences, as determined by Student’s t-test (*P < 0.05; **P < 0.01; two-tailed t test). **B)** The leaves of 4-week old Col-0 and *max2* mutant plants were inoculated with *P. syringae* and collected for analysis of SA level. The values are means ± SE of 2 independent experiments with 3 biological repeats in each experiment. Asterisks indicate significant differences, as determined by Student’s t- test (*P < 0.05; two-tailed t test).

To correlate the changes seen in stomatal phenotype and susceptibility to pathogens with possible alterations in endogenous hormone levels of *max2*, we measured the accumulation of ABA and SA (i) in non-stressed growth conditions, (ii) ABA-level after the leaves were excised and left to dry and (iii) (Figure 9A) and SA-level after *P. syringae* infection (Figure 9B). Interestingly, ABA levels in the *max2* mutant plants were higher already 30 min after excising the leaves and remained higher than in the leaves of wild-type plants until 4 h reflecting the increased water loss of *max2* plants (Figure 3A).

Both *P. syringae* and *P. carotovorum* trigger SA-dependent defense signaling in Arabidopsis [2,60,61]. Therefore, it was intriguing to determine, if the accumulation of endogenous SA was altered in *max2* plants in response to *P. syringae* and would contribute to the increased susceptibility of the plants. Interestingly, the only significant difference in pathogen-triggered SA-level between *max2* mutant and wild-type was 24 h after pathogen inoculation when the accumulation of SA was clearly higher in *max2* plants (Figure 9B). This could reflect the response of the plants to the dramatic increase in bacterial growth observed *in planta* at the same time (Figure 5B).

### Expression of SA related marker gene *PR1* is upregulated in *max2* plants

**Figure 10:**
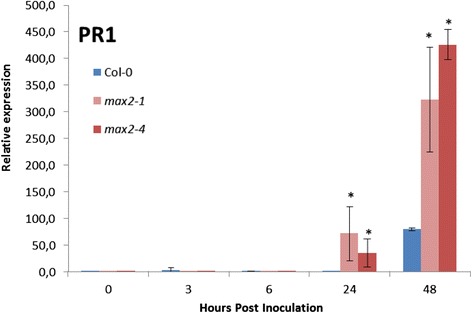
**The expression of SA related marker gene** ***PR1*** **is upregulated in** ***max2*** **lines in response to** ***P. syringae*** **.** Relative expression of *PR1* after *P. syringae* infection. Four-week old soil-grown plants were sprayed with *P.syringae* and samples collected at indicated time points for extraction of RNA. Asterisks indicate significant differences, as determined by Student’s t-test (*P < 0.05; two-tailed t test). For each experiment, 3 plants/line and 3 leaves/plant were used.

SA is known to contribute to the resistance to *P. carotovorum* and *P. syringae* [2,3]. Considering the decreased pathogen resistance of *max2* plants, in addition to the stomatal phenotype, the impact of possibly altered defense signaling could not be ruled out. To further explore the cause for the obvious decrease in plant resistance we characterized the expression of both SA- and JA-pathway marker genes in response to *P. syringae* infection. The expression of the marker gene for SA-dependent defense signaling, *PR1 (PATHOGENESIS-RELATED GENE1)* was significantly upregulated in wild-type plants 48 h after the spray inoculation. However, in *max2* mutant plants *PR1* was clearly induced already at 24 h and interestingly, at 48 h the expression of this gene was at least twice as high in *max2* as that observed in wild-type plants (Figure 10). The expression of *PR1* clearly indicates that the activation of SA-dependent defenses is enhanced in *max2* plants. Intriguingly, despite this *max2* plants are more susceptible to *P. carotovorum* and also *P. syringae* that should be contained by SA-mediated defense signaling.

While not central in defense to *P. syringae* or *P. carotovorum* in Arabidopsis, JA-dependent defense can still modulate the outcome of the interaction between these pathogens and Arabidopsis [2,61,62]. Therefore, in order to see if JA signaling was altered in *max2* lines and thus, would in its part influence the decreased resistance of these plants we examined the expression of JA-related marker genes *HEL (HEVEIN-LIKE)* and *VSP2 (VEGETATIVE STORAGE PROTEIN2)* [63] after inoculation with *P. syringae*. We could not observe any difference in the expression of these marker genes between *max2* and wild-type plants (data not shown) and conclude that JA does not contribute to the altered pathogen responses of *max2* plants.

## Discussion

There are over 700 F-box proteins in Arabidopsis the majority of which are still without an assigned function [25]. Since our interest lies in the characterization of plant response to environmental stresses, we wanted to identify yet uncharacterized F-box proteins with roles related to plant stress tolerance/disease resistance. We exposed several Arabidopsis F-box T-DNA insertion lines to ozone and the one with most distinct sensitive phenotype showing extensive tissue damage harbored the T-DNA insertion in *MAX2* gene (Figure 1). The F-box protein MAX2 (MORE AXILLARY GROWTH2), a negative regulator of polar auxin transport has earlier been shown to influence different processes, including strigolactone and karrikin signalling, auxin signaling and plant development, senescence, photomorphogenesis and responses to abiotic, such as drought and salt stresses in *Arabidopsis thaliana* [28,32,40,58,64-67]. Here, we further expand the role of MAX2 and provide evidence that it is also involved in biotic stress responses. Our results suggest that the increased susceptibility of *max2* plants to the phytopathogens *Pseudomonas syringae* and *Pectobacterium carotovorum* results from more open stomatal aperture and is further enhanced by decreased tolerance to stress-triggered apoplastic ROS and altered regulation of defense signaling.

Ozone enters plant cells via stomata and thus, triggers stomatal closure in the exposed plants [42]. Therefore, the increased ozone sensitivity of *max2* plants indicated alterations in the regulation of stomatal aperture of these plants. Indeed, measurements with both a porometer and a custom made gas-exchange device [50] indicated that the stomatal conductance of *max2* plants was higher than in wild-type in non-stressed conditions (Figures 3A and 4A). The altered stomatal phenotype is also supported by studies of Bu et al. 2014 [67] who, similarly to us (Figure 3B) show that the water loss from excised leaves is greater in *max2* than in wild-type plants. Interestingly, Ha et al. 2014 [58] observe no difference between the stomatal aperture of *max2* and wild-type in the absence of stress but show that the stomatal closure of *max2* plants in response to ABA is reduced. This is contradictory to our results showing that *max2* plants have wild-type like stomatal closure in response to ABA (Additional file 1: Figure S2). There could be several explanations for different results obtained for ABA responsiveness of *max2* mutants. We measured quick (up to 40 min) changes in stomatal conductance in response to single spraying of intact plants with 5 μM ABA, whereas Ha et al. 2014 [58] and Bu et al. 2014 [67] provide data about stomatal aperture changes in epidermal peels after 1–2 h of incubation in 10 μM ABA buffer. Additionally, also the density of stomata can influence the conductance. Whether this applies to *max2* plants remains to be solved: according to Ha et al. 2014 [58] *max2* mutants have increased stomatal density while Bu et al. 2014 [67] observe no such difference.

After entering the apoplast ozone degrades into O_2_^•–^ and H_2_O_2_, and causes the activation of NADPH oxidase leading to further ROS formation [68,69]. Thus, in addition to the increased intake, the ozone sensitivity of plants can also originate from impaired cellular responses to stress-triggered ROS. The excessive damage observed in the leaves of *max2* plants in response to ozone could indicate that the level of cellular ROS possibly exceeds the capacity of the plant antioxidant systems. This is further underlined by the rapid decrease in the amount of general photosynthesis measured in *max2* plants (Figure 4B). Also, the expression of ozone and ROS induced gene *GRX480* [55] was increased in *max2* indicating enhanced ozone response (Figure 7A). Moreover, the tolerance of *max2* lines to extracellular O_2_^•–^ generated by the xanthine/xanthine oxidase (X/XO) system was clearly decreased in comparison to that of wild-type plants (Figure 2). This suggests that the induction of extracellular O_2_^•–^ could trigger an ongoing production of ROS in *max2* lines that subsequently leads to increased damage.

Plant response to ozone and invading pathogens share some strikingly similar elements. Both ozone and bacterial pathogens enter the plant interior via stomata [6,42] and therefore, it was of great interest to test if the increased stomatal conductance observed in *max2* lines had any influence on the pathogen tolerance of the plants. Indeed, spray-inoculation with either the hemibiotroph *Pseudomonas syringae* or the necrotroph *Pectobacterium carotovorum* led to more severe disease development in *max2* lines compared to wild-type plants. Furthermore,when the plants were infected by applying the bacteria directly to apoplast (pipetting/infiltration) *max2* plants were still more susceptible but the difference to wild-type judging either by visual symptoms or bacterial numbers was significantly smaller in comparison to what was observed after spray inoculation (100 times more bacteria calculated in *max2* plants after spray-inoculation in comparison to infiltration) (Additional file 1: Figure S3). This strongly suggested that while other factors might also contribute, the more open stomatal aperture of *max2* plants has a central role in the increased susceptibility to both of these pathogens and that also the pathogen-triggered stomatal closure might be impaired in these plants. Indeed, this was confirmed when we observed that the well-described *P. syringae*-triggered stomatal closure described in Arabidopsis [54] was absent in *max2* plants (Figure 6). On the contrary, it seemed that instead of closure, *P. syringae* inoculation induced increase in stomatal opening in *max2* plants.

Similarly to ozone also pathogen invasion triggers apoplastic ROS burst originating from NADPH oxidase and peroxidases [42,44,70]. In pathogen responses, one function of the early produced ROS is its antimicrobial activity - it can be directly harmful to the invading pathogen [44,62]. However, the enhanced susceptibility of the *max2* plants did not suggest strongly increased ROS levels and accordingly, semiquantitative data on ROS accumulation did not show increase in either superoxide or H_2_O_2_ levels in these plants in response to pathogens or ozone (data not shown). ROS homeostasis is central in plants and for this, they possess a network of components of both ROS-producing and ROS-scavenging systems to secure appropriate ROS levels and at the same time minimize possible toxic effects of ROS [71,72]. Interestingly, despite the lack of increase in the pathogen-triggered ROS accumulation in the *max2* plants the induction of ROS-responsive *GSTI* and *GRNX480* genes triggered by *P. syringae* infection was clearly enhanced in comparison to wild-type (Figure 7B and C) indicating a stronger response to ROS. Also, the expression of auxin-responsive *HAT2* gene has earlier been shown to be downregulated by apoplastic ROS [55] and in comparison to wild-type, this gene is clearly less expressed in *max2* plants in response to both pathogens and ozone (Figure 7D and Additional file 1: Figure S6). This together with the ozone and X/XO-generated O_2_^•–^ -triggered damage in *max2* plants indicates that rather than enhanced accumulation, these plants might have decreased tolerance to ROS which further results in increased tissue damage.

However, after entry to the plant apoplast pathogen recognition triggers different lines of plant defenses, central of which is the activation of distinct defense signaling pathways mediated by different phytohormones such as SA, JA and ethylene [2,63]. The activation of these responses is further modulated by other phytohormones including auxin [2]. Auxin has for long been recognized as a central regulator of plant growth but its role as a modulator of plant defense responses to both abiotic and biotic stresses is getting more attention [16,19,66]. For example, it is well established that auxin and SA-mediated signaling are mutually antagonistic while auxin and JA signaling often seem to share synergism [13,16,19]. Modulation of auxin transport has been shown to influence activation of the SA dependent defenses. When the expression of a negative regulator of auxin transport, BUD1/MKK7 (BUSHY DWARF1/MAP KINASE KINASE7), was downregulated with RNAi the induction of SAR and resistance to pathogens was compromised in Arabidopsis [73]. Intriguingly, MAX2 is a negative regulator of polar auxin transport in Arabidopsis [31-33] and the increased expression of auxin response marker genes, such as *SAUR*s in *max2* plants [58] indicates enhanced auxin responses. Plant defense against both *P. carotovorum* and *P. syringae* in Arabidopsis is dependent on SA-signaling, and therefore, it was of interest to elucidate if the modulated auxin status of *max2* plants had any impact on the activation of SA-responses. Surprisingly, the only major difference between wild-type and *max2* plants in the expression of SA-dependent marker gene *PR1* was 24 and 48 h after *P. syringae* infection (Figure 10). There, especially at 48 h timepoint, the expression of *PR1* was clearly enhanced in *max2* plants, which was unexpected considering the increased susceptibility of these plants to *P. syringae*. However, at this point even the enhanced induction of SA-signaling is not enough to limit the massively spreading infection in *max2* plants.

Polar auxin transport is increased in *max2* plants which could be speculated to lead to downregulation of SA-responses similarly to *BUD1* mutant [74]. However, if the role of increased auxin transport had a major role in downregulating SA-response for example during the early hours of infection in *max2* plants thus increasing the susceptibility then higher expression of *PR1* should have been observed in wild-type plants and this was not the case (Figure 10). At this time the reason for increased SA accumulation and enhanced *PR1* expression observed 24 and 48 h after bacterial inoculation in *max2* lines remains elusive.

Based on our results, we show that MAX2 contributes to biotic stress resistance in Arabidopsis and thus, expand the already established role of MAX2 in developmental and abiotic stress responses. Resistance to the phytopathogens *P. carotovorum* and *P. syringae* is clearly compromised in *max2* mutants*.* We propose that the decreased resistance of *max2* plants results mainly from more open stomatal aperture but is further accentuated by the decreased tolerance of these plants to stress-triggered ROS and by altered defense signaling possibly influenced by enhanced auxin responses. Indeed, auxin has been shown to promote stomatal opening and thus, enhance the progression of the disease [12-14].

The exact molecular mechanism how MAX2 mediates these events requires more detailed molecular studies, for example identification target proteins and thus, remains a subject for future studies.

## Conclusions

Our results show that MAX2 previously characterized for its role in the regulation of polar auxin transport and thus, plant development in Arabidopsis also significantly influences plant disease resistance. Our data reveals that increased susceptibility of *max2* plants to *Pseudomonas syringae* and *Pectobacterium carotovorum* is due to increased stomatal conductance leading to enhanced pathogen entry into the plant apoplast. Moreover, *max2* plants were shown to have decreased tolerance to apoplastic ROS showing that MAX2 is also required for the regulation of ROS-induced cell death at mesophyll level. The activation of defense signaling in response to pathogens is also altered in *max2* plants, presumably resulting from perturbations in the auxin homeostasis of these plants.

## Methods

### Plants and growth conditions

*Arabidopsis thaliana* ecotype Col-0 wild type and mutant plants were grown in a growth room using 1:1 peat:soil mixture (Finnpeat B2; Kekkilä Oyj) with a 12 h light period at 22°C. Approximately 1 week after germination, individual seedlings were transferred to grow in soil. All mutants used in this study are derived from Col-0. *max2-1* (point mutation line described by Stirnberg et al. 2002 [28]) and *max2-4* (SALK_028336) were obtained from the Salk Institute (http://signal.salk.edu/cgi-bin/tdnaexpress) and *rcd1-4* (At1g32230) was obtained from Jaakko Kangasjärvi (University of Helsinki). Plants used for oxidative stress and pathogen stress tests were 4 weeks old and 4–5 weeks old for porometer measurements and water loss tests.

### Ozone treatment

The ozone exposure was conducted in a growth chamber with a concentration of 300 nL L^−1^ or 350 nL L^−1^ of ozone continuing for 6 h as described in Overmyer et al. 2000 [42].

### Pathogen infections and stress treatments

Plants were infected with two bacterial pathogen strains, *Pectobacterium carotovorum* subsp*. carotovorum* SCC1 and *Pseudomonas syringae* pv. *tomato* DC3000 and a necrotrophic fungal pathogen, *Botrytis cinerea* Pers.: Fr strain B.05.10 [75]. *P. carotovorum* was propageted in Luria medium [76] at 28°C. The bacteria were collected by centrifuging 4000 rpm for 2 min and washed with 50 mM NaCl. Centrifugation and washing were repeated and the bacteria was suspended in 50 mM NaCl. In the infection solution amount of bacteria was adjusted to 1 × 10^5^ cfu/ml. A leaf was wounded with a pipette tip and 10 μl of bacterial solution was applied to the wound site. The plants were covered with plastic to keep the moisture high and scored for symptom development 24 h and 48 h post infection. The amount of bacteria used for spray infection was 1 × 10^6^ cfu/ml. Silwet L-77 (0,02%) was added in solution just before the infection to reduce surface tension.

*P. syringae* was propagated in King’s B media at 28°C. The bacterial cells were collected by centrifuging 6000 rpm for 8 min and washed with 10 mM MgCl_2_. The centrifugation was repeated and the bacteria was resuspended in 10 mM MgCl_2_. Bacterial concentration of 1 × 10^6^ cfu/ml used for infiltration and 1 × 10^7^ cfu/ml used for spray inoculation. In infiltration experiments, approximately 10 μl of bacterial suspention used and at indicated time points 0.5 cm^2^ leaf disc at the site of infection were harvested and the number of viable bacteria in each disc was determined. For spray infection, Silwet L-77 (0,02%) was added in solution just before the infection to reduce surface tension. Plants were covered well to provide enough humidity for successful infection.

Prior to use, *B. cinerea* was subcultured on potato carrot agar plates (PCA). The plates were kept for 6–8 d in darkness and then placed on lab bench for sporulation at room temperature for a few days, then kept in cold room (+4°C) until infection time. Conidia were harvested from 14-d-old cultures by scratching with 10 ml inoculation medium (Potato dextrose broth-PDB). Liquid medium with spores were vortexed 15 min then filtered through cheesecloth. For the experiment the inoculum concentration was adjusted to *c.* 1 × 10^5^ conidia ml^−1^. For inoculation a 10 μl droplet was placed on the upper surface of the leaf and three leaves per plant were infected. Infected plants were kept inside plastic boxes covered with a clear plastic wrap and added enough amount of water to provide high humidity for 3 days.

### Stomatal response to incubation with bacteria

Stomatal response to bacterial infection was done based on a method developed by Chitrakar and Melotto 2010 [54]. Briefly, plant leaves were stained first in 20 μM propidium iodide (PI) solution for 5 min then placed on a microscope slide lower surface facing dawn. Then 300 μL bacterial solution concentraion of OD 0.2 corresponding to 10^8^ CFU/mL added and incubated in same growing condition as plants grown before. For the time points measured, leaves transferred on a different microscope slide and placed lower surface facing up. To examine the leaves, OLYMPUS BX63 fluorescent microscopy is used. Leaf samples were imaged and the aperture width of between 80 to 100 stomata for each treatment at each time point, were measured using ImageJ image prossessing program.

### Xanthine + xanthine oxidase treatment

8 mm leaf disks were cut from 4–5 week old plants and floated in nonautoclaved MQ water to wash. 4 disks per replicate × 4 replicates were used for every line. The disks were placed into 5 ml of 10 mM sodium phosphate buffer (pH 7.0) with 1 mM xanthine. Xanthine oxidase (Sigma-aldrich) (0.05 Unit/ml final concentration) was quickly added, swirled to mix and vacuum infiltrated. The X/XO infiltrated tubes were incubated on lab bench for 4 h, production of superoxide will be over after 3–3.5 h. After incubation the buffer was poured off and the disks were washed with non-autoclaved MQ water. The leaf disks were transferred to a tube containing 6 ml non-autoclaved MQ water and the conductivity measured after 0, 1, 2, 4, 8, 12 and 24 h.

### Methyl viologen assays

Sensitivity of germination to methyl viologen was assessed using ½ MS plates containing 0.05 or 1 μM of methyl viologen (Sigma-Aldrich). Sterilized seeds of *max2-1*, *max2-4, rcd1-4* and Col-0 were germinated with a 12 h low light period at 22°C. The root length was measured 2 weeks after germination.

### ROS staining assays

For visualizing the amount of H_2_O_2_, detached leaves were vacuum infiltrated with 0.1% DAB (Diaminobenzidine tetrahydrochloride, Sigma-Aldrich) in 10 mM MES (2-(N-morpholino)ethanesulfonic acid), pH 6.5 for 30 min. For O_2_^•–^ staining detached leaves were first infiltrated with K_2_PO_4_ buffer (pH 7.8) for 30 min and then the buffer was changed into 0.1% NBT (Nitroblue tetrazolium, Sigma-Aldrich) in 10 mM K_2_PO_4_ buffer and the leaves were further vacuum infiltrated for 30 min. After both stainings the leaves were cleaned by boiling in alcohol-lactophenol (2:1) for 5 min, and then rinsed twice with 50% ethanol and once with MQ water.

### Stomatal conductance and water loss

Stomatal conductance was measured with an AP4 Porometer (Delta-T Devices, Cambridge, UK). Two leaves were analysed from five different individual plants for each genotype and the experiment was repeated with similar results five times.

For water loss measurements according to Leung et al. 1997 [77]; three detached rosette leaves of *max2-1*, *max2-4* and wild-type Col-0 plants were incubated abaxial face up at ambient laboratory conditions. Fresh weight of the detached leaves was measured at various time points for 240 min. Water loss was expressed as percentage of initial fresh weight upon excision. Five individual plants were used for each genotype and the experiment was repeated with similar results five times.

Stomatal responses to exogenous ABA (Additional file 1: Figure S2) and to 3 h 350 nmol/mol ozone exposure were measured with the custom made eight-chamber whole-plant rapid-response gas exchange measurement device described previously [50]. Standard conditions during the stabilization were: ambient CO_2_ (c. 400 ppm), light 150 μmol m^−2^ s^−1^, RH 60–70%. Distilled water with 0,012% Silwet L-77 (Duchefa) and with or without 5 μM of abscisic acid was sprayed onto intact Arabidopsis rosettes, air-dried in the measurement cuvette and 7 min after spraying stomatal conductance was recorded for the next 40 min.

### Real-time quantitative PCR analysis

RNA was isolated using GeneJET Plant RNA Purification Mini Kit (Thermo Scientific). Total RNA was treated with DNase I (Thermo Scientific) and 0.5-1 μg was used for the synthesis of cDNA. For cDNA synthesis, Maxima Reverse Transcriptase (Thermo Scientific) and Ribolock RNase inhibitor (Fermentas) were used according to the manufacturer’s instructions. For each qRT PCR reaction 8 ng of cDNA was used as a template and the reaction was performed using Solis BioDyne HOT FIREPol EvaGreen qPCR Mix Plus (no ROX) on a Bio-Rad CFX384. The results were calculated using Biogazelle’s qBase qPCR program based on geNorm technology. In pathogen experiments three reference genes TIP41, PP2AA3 and At5g15710 were included and validated to have a stable expression. In ozone experiments TIP41 and PP2AA3 were used as reference genes and validated to have a stable expression. The sequences of all the primers used in real-time quantitative PCR are included in Table 1.

**Table 1:** **Primer lists**

**Gene name**	**AGI code**	**Primer sequence**
At1g13320	PP2AA3	GCGGTTGTGGAGAACATGATACG
At1g13320	PP2AA3	GAACCAAACACAATTCGTTGCTG
At4g34270	TIP41	GTGAAAACTGTTGGAGAGAAGCAA
At4g34270	TIP41	TCAACTGGATACCCTTTCGCA
At5g15710	F-box	GGCTGAGAGGTTCGAGTGTT
At5g15710	F-box	GGCTGTTGCATGACTGAAGA
At3g62980	TIR1	GCATTTGCAGGAGACAGTGA
At3g62980	TIR1	AAACGGGCAGTCCCTTATCT
At4g03190	AFB1	GGGGACAGTGATTTGATGCT
At4g03190	AFB1	TGTCTCCAAAAGGGCAGTCT
At5g47370	HAT2	CGAACCATCACCACAATCAC
At5g47370	HAT2	GCAAGGCTTCAAAATTCAGC
At1G28480	GRX480	ACGGAGAGGATGTTGCATGTGTC
At1G28480	GRX480	AATCTCAAGGACCGCCGGATTC
At1G02930	GST1	CAAGGACATGGCGATCATAGC
At1G02930	GST1	TCCCAAACAAGCTTTGAACCA
At2g14610	PR1	CGGAGCTACGCAGAACAACT
At2g14610	PR1	CTCGCTAACCCACATGTTCA

### Phytohormone measurements

Approximately 100 mg of fresh plant material was weighed, immediately frozen in liquid nitrogen and ground with a ball mill (Retsch, Haan, Germany) in 2 ml Eppendorf tubes. Phytohormones were extracted and analyzed with a Waters Synapt GS HDMS mass spectrometer (Waters, Milford, MA, USA) interfaced a Waters Acquity UPLC® system (Waters, Milford, MA, USA) via a negative electrospray ionization (ESI) source as described in Li et al. 2013 [9].

## Additional file

Additional file 1: Figure S1. Effect of Methyl viologen on root elongation assay of Col-0, *rcd1* and *max2* mutant lines. **Figure S2.** *max2* mutant lines close their stomata normally in response to ABA treatment. **Figure S3.** *max2* mutant lines do not have altered resistance after bacterial application by infiltration/pipetting. **Figure S4.** Wild-type Col-0 and *max2* lines show similar phenotype to *Botrytis cinerea*. **Figure S5.** Treatment of *max2* and wild-type leaves with MgCl_2_ buffer solution. **Figure S6.** *HAT2* expression after ozone treatment.

## Abbreviations

ABA: Abscisic acid
SA: Salicylic acid
ET: Ethylene
PAMP: Pathogen-associated molecular pattern
JA: Jasmonic acid
ROS: Reactive oxygen species
X/XO: Xanthine/xanthine oxidase
DAB: Diaminobenzidine tetrahydrochloride
NBT: Nitroblue tetrazolium
