# Supplementary material for: The F-box protein MAX2 contributes to resistance to bacterial phytopathogens in Arabidopsis thaliana
Source: BMC Plant Biol. 2015 Feb 13;15:53. doi: 10.1186/s12870-015-0434-4 (PMC4340836; doi:10.1186/s12870-015-0434-4)
Supplement: Additional file 1: Figure S1. — Effect of Methyl viologen on root elongation assay of Col-0, rcd1 and max2 mutant lines. Figure S2. max2 mutant lines close their stomata normally in response to ABA treatment. Figure S3. max2 mutant lines do not have altered resistance after bacterial application by infiltration/pipetting. Figure S4. Wild-type Col-0 and max2 lines show similar phenotype to Botrytis cinerea. Figure S5. Treatment of max2 and wild-type leaves with MgCl2 buffer solution. Figure S6. HAT2 expression after ozone treatment. [file 12870_2015_434_MOESM1_ESM.docx]

**Additional file**

**Additional file 1 FigureS1 Effect of Methyl viologen on root elongation assay of Col-0, *rcd1* and *max2* mutant lines.** In-vitro grown 2 weeks old Col-0 and *max2-1* point mutation lines’ root lengths were measured after 1 and 2 µM methyl viologen treatments. *rcd1-4* is known for its tolerance to methyl viologen and therefore used as a control. Asterisks indicate significant differences, as determined by Student’s t-test (** P < 0.01; two-tailed t test).


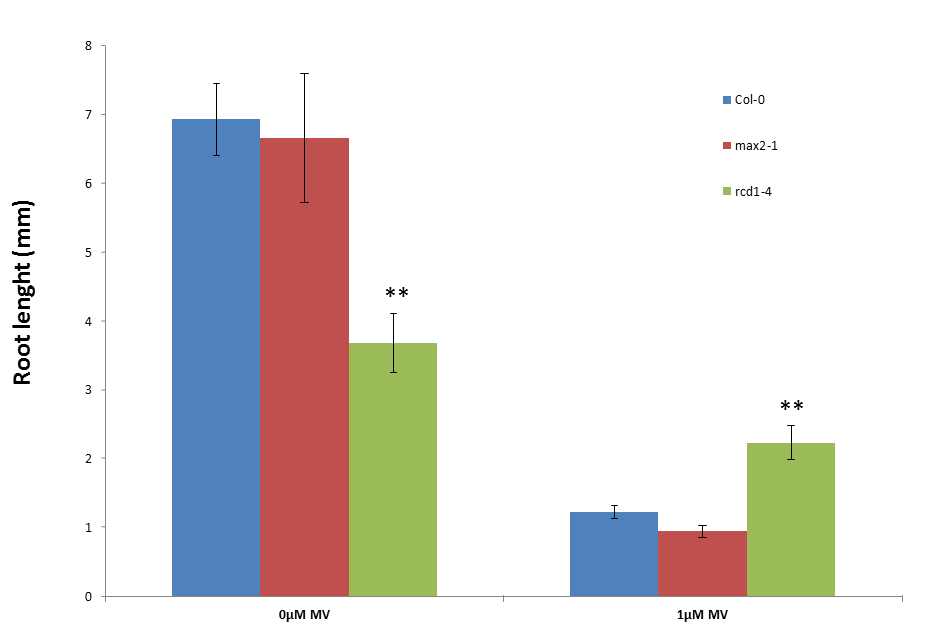


**Additional file 1 FigureS2 *max2* mutant lines close their stomata normally in response to ABA treatment.** Stomatal conductance in absolute (A) and relative units (B) of Col-0 and *max2* intact plants sprayed with 5 µM ABA solution at time point 0 (indicated by arrow). Spraying with 5 µM ABA induced similar stomatal closure in WT Col-0 and *max2* mutants. For each line altogether 8-9 plants from two separate repeats were used and the results are shown as means ± SE. C) Stomatal conductance in absolute units of Col-0 and *max2* intact plants sprayed with mock solution at the time point 0. No stomatal response to the spraying could be observed. For each line 3-5 plants from were used and the results are shown as means ± SE.


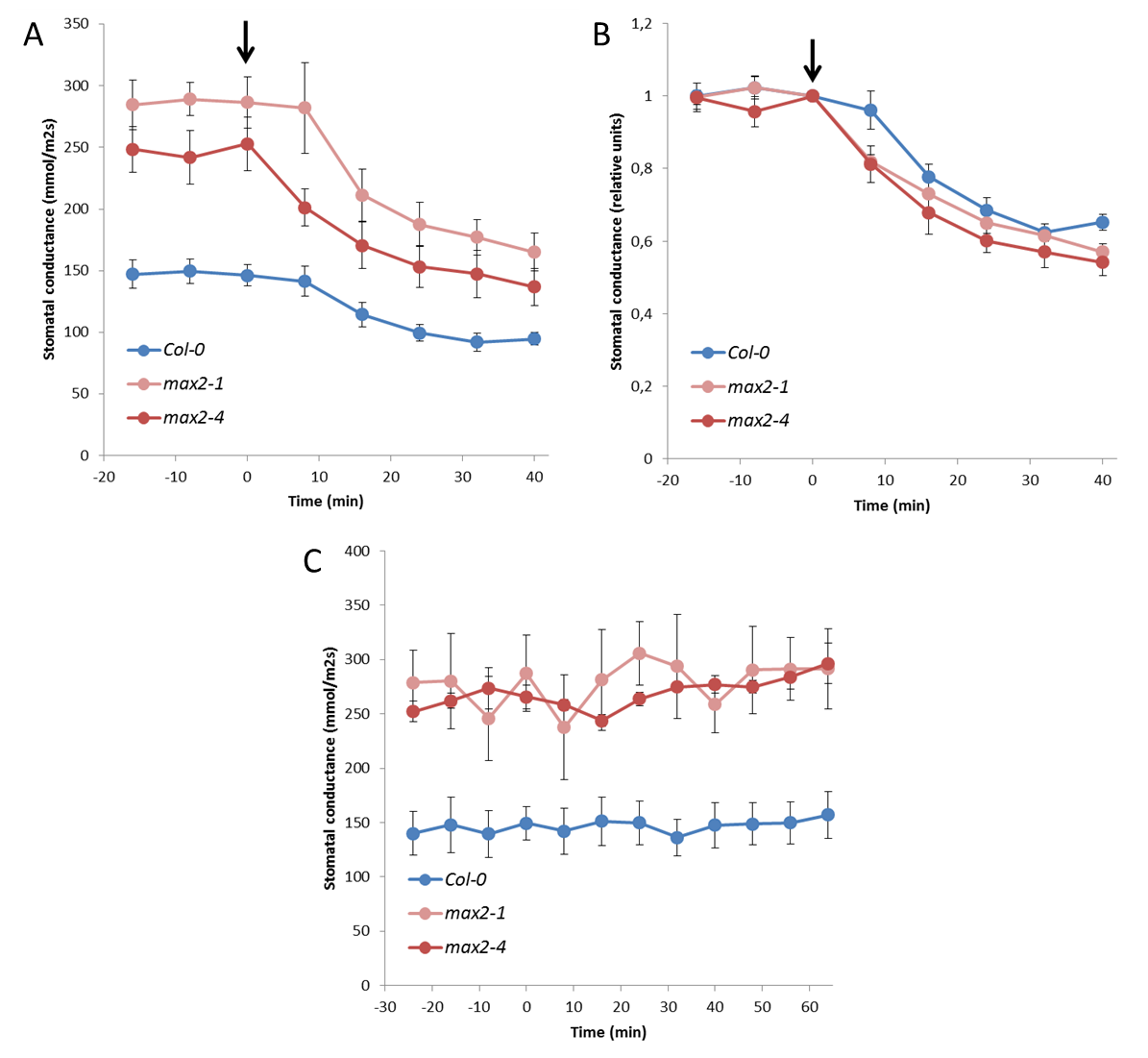


**Additional file 1 FigureS3 *max2* mutant lines do not have altered resistance after bacterial application by infiltration/pipetting.** A) Four-week old soil-grown plants were infiltrated with approximately 10 µl of bacterial suspension containing 10^6^cfu/ml. Three plants/line and three leaves/plant used in each experiment to measure the bacterial concentration and experiment repeated three times with similar results. At the indicated times, 0.5 cm^2^ leaf disc at the site of infection were harvested and the number of viable bacteria in each disc was determined. The results are shown as means ± SE (**P < 0.01; two-tailed t test). B) 4-week old soil grown Col-0 and *max2* plants were infected with *P.carotovorum*. First, a small wound made on a plant leaf and then 10 µl of bacterial solution applied by pipetting. Asterisks indicate significant differences, as determined by Student’s t-test (* P < 0.05; ** P < 0.01; two-tailed t test).


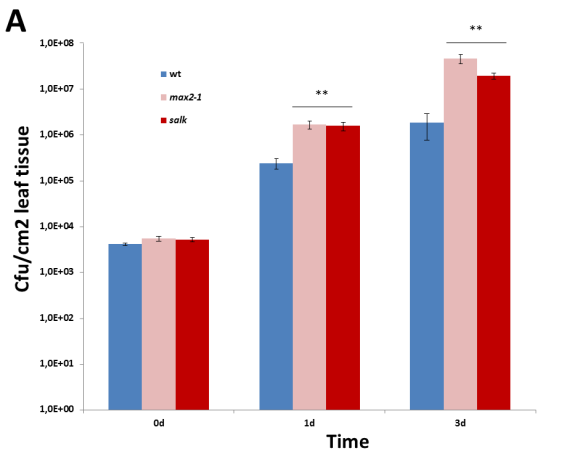

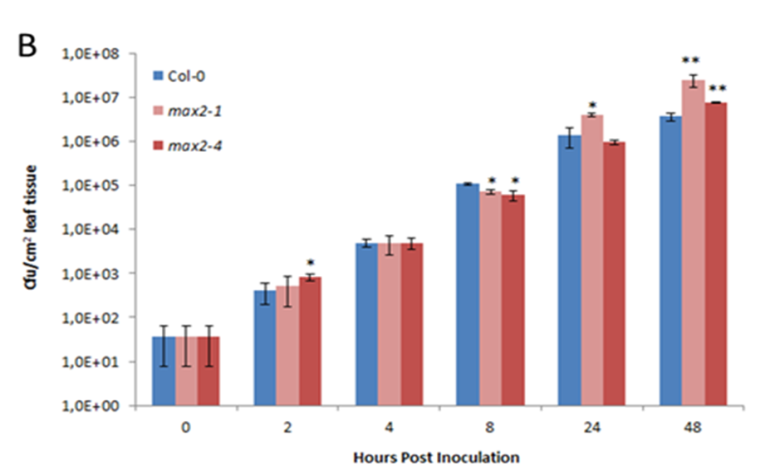


**Additional file 1 Figure S4 Wild-type Col-0 and *max2* lines show similar phenotype to *Botrytis cinerea*.** 10 µl droplets of a suspension of *B. cinerea* spores (1x10^5^ spores ml^-1^) were placed on a 4-week-old plant leaves. Image show the phenotype of wild-type Col-0 and *max2* lines 3 d after inoculation. Experiment repeated several times with same results.


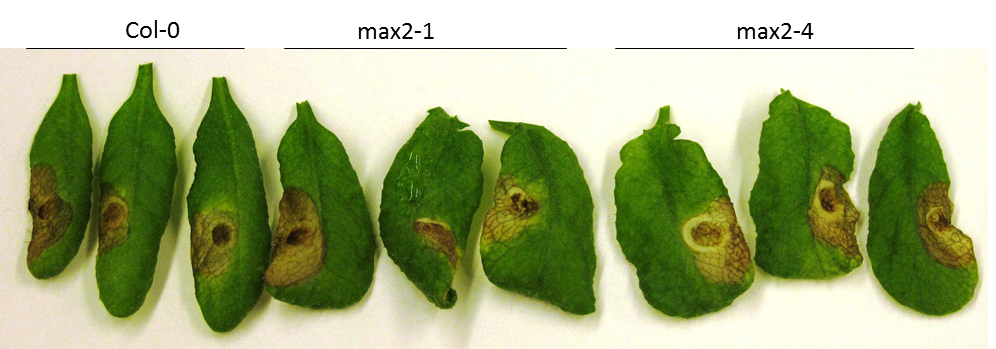


**Additional file 1 Figure S5 Treatment of *max2* and wild-type leaves with MgCl_2_ buffer solution.** Four-week old plant leaves first stained with 20 µM propidium iodide solution for 5 min. Following staining 300 µl 10 mM MgCl_2_ buffer solution added and stomatal response checked at different time points to see if buffer solution causes the observed phenotype. To examine the leaves, OLYMPUS BX63 fluorescent microscopy is used. Leaf samples imaged and the aparture width of between 80 to 100 stomata at each time point measured using ImageJ image prossessing program.


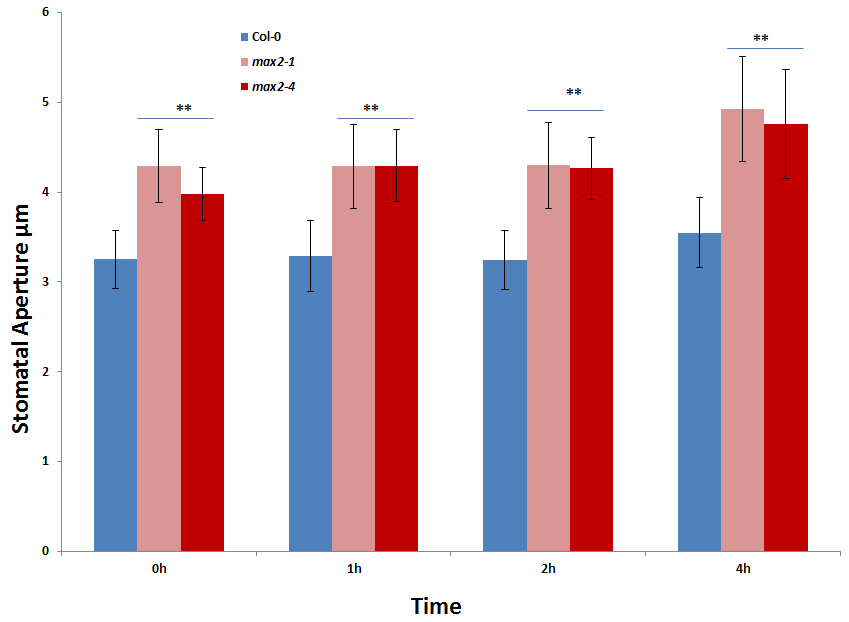


**Additional file 1 Figure S6 *HAT2* expression after ozone treatment.** Mature leaves of 4-week old soil grown wild-type Col-0 and *max2* plants were collected at indicated time points of ozone exposure (350 ppb of ozone for 6 hours) and expression of auxin-responsive gene *HAT2* was analysed. 3 plants/line and 3 leaves/plant were used in each time point of ozonation.
